# Sustaining Robust Cavities with Slippery Liquid–Liquid Interfaces

**DOI:** 10.1002/advs.202103568

**Published:** 2022-01-17

**Authors:** Suwan Zhu, Tao Wu, Yucheng Bian, Chao Chen, Yiyuan Zhang, Jiawen Li, Dong Wu, Yanlei Hu, Jiaru Chu, Erqiang Li, Zuankai Wang

**Affiliations:** ^1^ CAS Key Laboratory of Mechanical Behavior and Design of Materials Hefei National Laboratory for Physical Sciences at the Microscale Laboratory of Precision Scientific Instrumentation of Anhui Higher Education Institutes Department of Precision Machinery and Precision Instrumentation University of Science and Technology of China Hefei 230026 China; ^2^ Department of Modern Mechanics University of Science and Technology of China Hefei 230026 China; ^3^ Department of Mechanical and Biomedical Engineering City University of Hong Kong Hong Kong 999077 China; ^4^ Shenzhen Research Institute of City University of Hong Kong Shenzhen 518057 China

**Keywords:** cavity formation, drag reduction, droplet impact, slippery surfaces, water entry

## Abstract

The formation of a stable gas cavity on the surfaces of solid bodies is essential for many practical applications, such as drag reduction and energy savings, owing to the transformation of the originally sticky solid–liquid interface into a free‐slip liquid–vapor interface by the creation of either liquid repellency or a Leidenfrost state on the surfaces. Here, it is shown that the simple infusion of a textured sphere with a smooth, slippery liquid layer can more easily create and sustain a stable gas cavity in a liquid at lower impact velocities compared to a dry solid sphere with the same contact angle. With a key parameter of curvature ratio, the early lamella dynamics during water entry of spheres and drops impact on planes are first unified. With the perspective of wetting transition, the unforeseen phenomenon of prone to cavity formation are successfully explained, which is the preferential lamella detachment from a slippery surface due to the higher viscosity of the lubricant relative to air. It is envisioned that the findings will provide an important and fundamental contribution to the quest for energy‐efficient transport.

## Graphical abstract

The simple infusion of a textured sphere with a smooth, slippery liquid layer (termed as SLIPS sphere) can more easily create a stable gas cavity in water at lower impact velocities compared to a dry hydrophobic sphere with the same contact angle, due to the preferential water lamella detachment from the slippery surface.

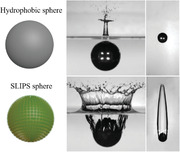

## 1 Introduction

The entry of solid bodies into liquids is widely manifested in daily life and industrial activities.^[^ ^1^, ^2^, ^3^, ^4^, ^5^, ^6^, ^7^, ^8^ ^]^ Generally, the formation of a continuous and sustained gas cavity surrounding solid surfaces that rectifies the triple interface into a smooth liquid‐gas interface is highly preferred because of many advantages, such as drag reduction and modulation of heat transfer.^[^ ^9^, ^10^, ^11^, ^12^, ^13^, ^14^ ^]^ To maintain the gas cavity, two approaches have been widely developed: either the use of external heating^[^ ^15^, ^16^, ^17^, ^18^, ^19^ ^]^ or the design of hydrophobic surfaces.^[^ ^6^, ^14^ ^]^ For the former, when the solid surface temperature drops due to heat transfer to the surrounding liquid, the gas cavity is susceptible to collapse.^[^ ^10^ ^]^ For the latter, efforts have been made to achieve surfaces with superrepellency for a wide range of liquids^[^ ^20^, ^21^, ^22^ ^]^ and mechanical robustness.^[^ ^23^ ^]^

Inspired by the unique surfaces of Nepenthes pitcher plants in nature, lubricated surfaces consisting of micro/nanostructures impregnated with low‐surface‐energy oils (slippery liquid‐infused porous surfaces, termed SLIPSs or LISs) were proposed and investigated, featuring extremely low contact angle hysteresis (<5°) and excellent repellency for foreign liquids on the surfaces.^[^ ^24^, ^25^ ^]^ Regarding practical uses, SLIPSs are frequently reported in a number of potential applications, such as in drag reduction,^[^ ^26^, ^27^ ^]^ anti‐icing,^[^ ^28^, ^29^ ^]^ and anti‐fouling.^[^ ^30^ ^]^

The major merits of SLIPSs of exceptional liquid repellency and minimized interfacial friction inspire us with new insights and physics for forming robust gas cavities. Compare to a hydrophobic sphere, we show that it is easier for a SLIPS sphere with the same water contact angle to produce a sustained, robust gas cavity during water entry.

## 2 Results and Discussion

**Figure 1:**
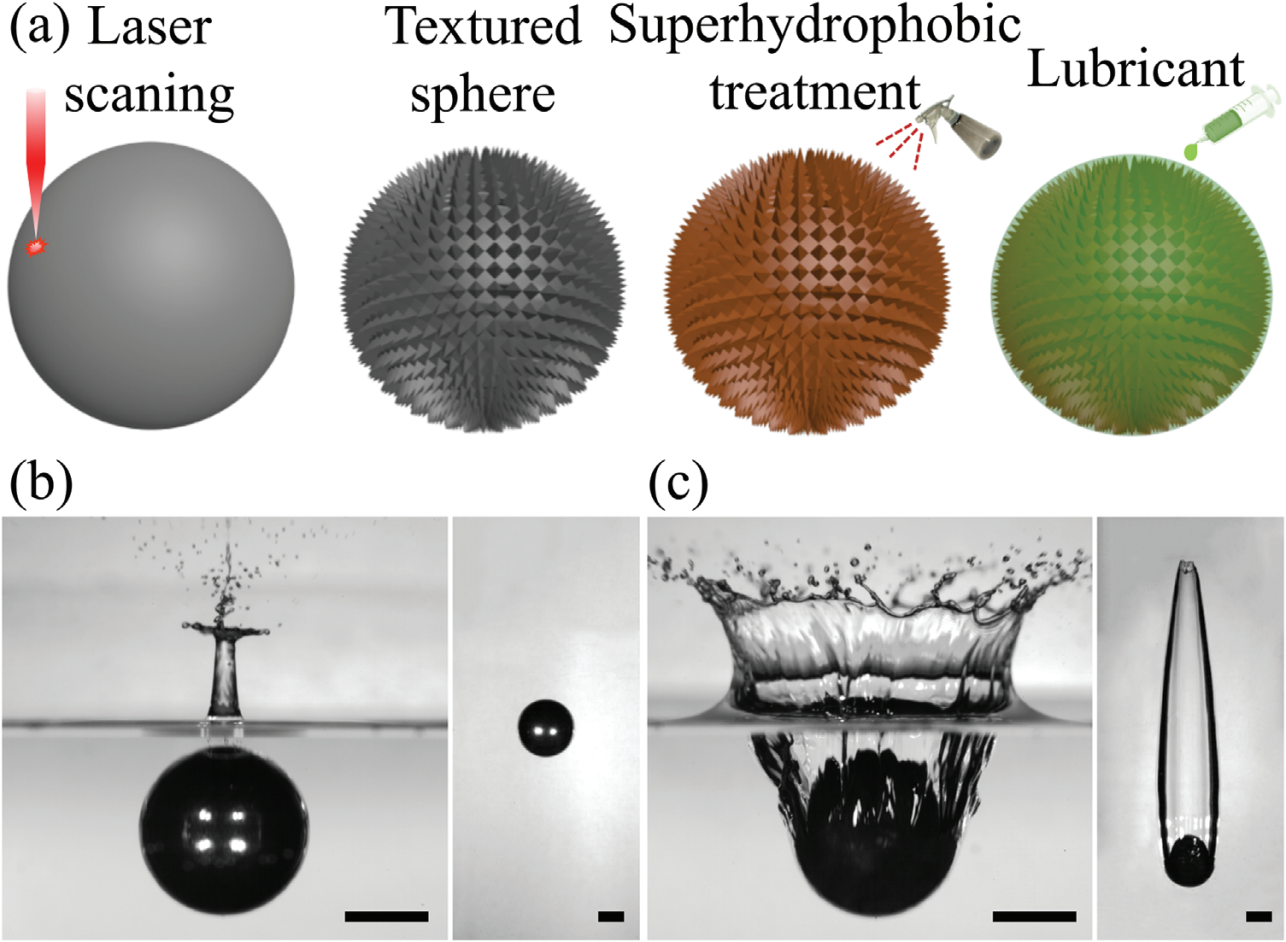
Distinct water entry behaviors between hydrophobic and SLIPS spheres. a) Schematic illustration of the SLIPS sphere fabrication process. b) Water entry of a hydrophobic sphere at an impact velocity of *U* = 3.0 m s^−1^. The right side of the figure shows a wide view of the sphere underneath the water surface. No cavity is produced for this hydrophobic sphere. c) Water entry of a SLIPS sphere at *U* = 3.0 m s^−1^. The right side of the figure shows the steady‐state gas cavity formed around the SLIPS sphere. The scale bars are 5 mm. Here, the equilibrium water contact angle on hydrophobic spheres is 110°, the same as that on SLIPS spheres.

As illustrated in **Figure** **1**a, to fabricate a SLIPS sphere, we first constructed textures on a steel sphere (density *ρ* = 7800 kg m^−3^) with a radius *r*_1_ = 0.5 cm by femtosecond laser irradiation. The laser‐textured sphere surface consists of micro/nanostructures whose morphology can be tailored by controlling the laser scanning speed (Figure S1, Supporting Information). In this study, the average distance between the adjacent micropillar‐like structures on the textured sphere was ≈25 µm. The textured sphere was hydrophobized by immersing it into 3 wt% 1H, 1H, 2H, 2H‐perfluorodecyltriethoxysilane (CAS No. 101947‐16‐4) in ethanol (CAS No. 64‐17‐5) solution for 24 h and then heated at 120 °C for 30 min, followed by infusion of a certain amount of perfluorinated fluid (3M Fluorinert FC‐3283) onto its surface. Hydrophobizing the textured sphere will lead to a more stable lubricant film.^[^ ^24^ ^]^ The lubricant thickness was calculated by dividing the volume of the lubricating fluid added to the textured sphere over the sphere surface area, the same way as used in literature.^[^ ^24^ ^]^ Here, the perfluorinated fluid acts as a lubricant film, and its physical properties are density *ρ*_f_ = 1830 kg m^−3^, dynamic viscosity *μ*_f_ = 1.5 mPa s and surface tension *γ*_f_ = 16.0 mN m^−1^ at 20 °C. Later, we will show that *μ*_f_ is an important parameter governing cavity formation.

The experimental configuration is shown in Figure S3, Supporting Information. The hydrophobized sphere was attached to an electromagnetic release system positioned directly above the center of a water tank. After adding the lubricant to the hanging sphere via a pipette, the sphere was released, and the rapid surface motions during the impact were recorded by a high‐speed camera at frame rates up to 110 kfps. The lubricant thickness was found to be an important parameter for liquid repellency on a SLIPS film, as evidenced by a dramatic increase in the apparent contact angle hysteresis or even the occurrence of droplet pinning when the lubricant liquid was insufficient to cover the surface textures^[^ ^24^ ^]^ (in their Figure S4, Supporting Information).

To eliminate the effects of lubricant film thickness on contact angle hysteresis,^[^ ^24^ ^]^ in this study, sufficient lubricant was added to fully cover the solid sphere. The lubricant evaporation rate was evaluated by measuring the mass loss from a planar SLIPS surface, which gave a decrease in lubricant film thickness of ≈0.3 µm s^−1^ (Figure S4, Supporting Information) at a room temperature of 20 °C. Therefore, lubricant evaporation is negligible during the experimental operation. The equilibrium water contact angle on the SLIPS sphere is ≈110°. The elegant work of Duez et al.^[^ ^14^ ^]^ showed that the wettability of the sphere is a key factor affecting splashing and the threshold velocity for cavity formation reduces when the static contact angle increases for hydrophobic spheres. Therefore, compared to a SLIPS sphere here with a water contact angle of 110°, a superhydrobic sphere will require a lower threshold velocity to produce a cavity. For comparison, hydrophobic spheres with the same contact angle were fabricated with an electronic grade coating (3M Novec 1700) and tested (Figure S2, Supporting Information).

**Figure 2:**
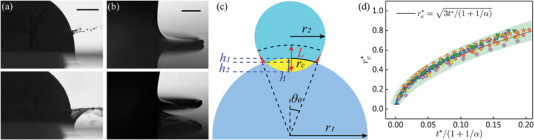
Early lamella evolution for water entry of hydrophobic/SLIPS spheres and water droplets impacting hydrophobic/SLIPS planes and spheres. a) The lamella climbed up along the hydrophobic sphere surface, while it detached from the SLIPS sphere for the same sphere impact velocity of 2.0 m s^−1^. Shown at time *t* = 947µs relative to first contact. The scale bar is 2 mm. b) The lamella spread along the hydrophobic plane, while it detached from the SLIPS plane and invaded air for the same droplet impact velocity of 2.0 m s^−1^ and droplet radius *r*_2_ = 1.75 mm. Shown at time *t* = 382µs relative to first contact. The scale bar is 250 µm. c) Schematic of droplets impact on spheres. *r*_c_ = *θ*_0_ *r*_1_ is the half arc length of the wetted spherical cap. d) The radius of the contact line varied with time and radius ratio α. ■: *α* = 5.38, •: *α* = 2.86, ▲: *α* = 1.61, ▼: *α* = 0.86, ✦: *α* ≈ ∞, and ★ represents the water droplet impact on 0.65 cSt silicone oil‐infused surfaces.

Interesting results were obtained when comparing the different entry dynamics of hydrophobic and SLIPS spheres at gradually elevated impact velocities *U* (Movie S1, Supporting Information). First, although both spheres exhibit the same equilibrium water contact angle, the threshold impact velocities of cavity formation for the SLIPS and hydrophobic spheres were 2.0 and 4.5 m s^−1^, respectively, which were significantly different. Figure 1b,c shows that at *U* = 3.0 m s^−1^, a gas cavity was formed during the water entry of a SLIPS sphere, whereas no gas cavity was produced around the hydrophobic sphere. In striking contrast to the previous perception that wettability is a key parameter in determining gas cavity formation,^[^ ^6^, ^14^ ^]^ our experimental results suggest a different picture. Second, the advancing film, termed as lamella, exhibits quite different configurations for the two spheres. As shown in **Figure** **2**a and Movie S2, Supporting Information, at *U* = 3.0 m s^−1^, the lamella climbed up along the hydrophobic sphere surface and eventually merged at the north pole of the sphere. In contrast, on the SLIPS sphere, the lamella detached from the sphere body and created an evident crown‐like structure, which led to the formation of a stable gas cavity after crown closure.

The lamella evolution during impact is critical to cavity formation, but remains challenging to visualize in detail for the water entry configuration, as the water surface severely deforms and obstructs the view. Herein, we elaborately designed a series of droplet impact experiments on planes and spheres where similar crown‐like structures were observed, and the lamella morphology at the earliest stage could be clearly visualized, as shown in Figure 2b. To build a link between the water entry of spheres and droplet impact, we defined a radius ratio α = *r*_1_/*r*_2_ for a droplet‐sphere collision system, where *r*_1_ is the radius of the solid sphere and *r*_2_ is the radius of the droplet, as shown in Figure 2c. Particularly, *α* = 0 corresponds to the water entry of spheres, and *α* = ∞ corresponds to droplet impact on a plane. In the early stage of impact, the liquid volume cut by the sphere (the yellow part in Figure 2c) must radially outstretch.^[^ ^31^ ^]^ From the geometric relationship, we obtain $r_{1}^{2}-{\left(r_{1}-h_{1}\right)}^{2}=r_{2}^{2}-{\left(r_{2}-h_{2}\right)}^{2}$ and *h*_1_ + *h*_2_ = *h*. The volume of a spherical cap of height *x* from a sphere of radius *r* is *V*_cap_(*r*, *x*) = π*x* ^2^(3*r* − *x*)/3. Here, we define the intersection of two spheres as a multiple spherical cap, the volume of which is *V*_mcap_(*r*_1_, *r*_2_, *h*) = *V*_cap_(*r*_1_, *h*_1_) + *V*_cap_(*r*_2_, *h*_2_). Apparently, the volume of the yellow part in Figure 2c is *V*_I_ = *V*_mcap_(*r*_1_, *r*_2_, *h*), and the volume of the red part is $V_{\text{II}}=\pi \theta_{0}^{2}r_{1}^{2}\left(L-h\right)-\left[V_{\text{mcap}}\left(r_{1}+L-h,r_{2},L\right)-V_{I}\right]$. Volume conservation gives *V*_I_ = *V*_II_, and for the very early stage of impact, we have *h* ≪ *r*_1_, $\theta_{0}\approx \sqrt{L/[\left(1+\alpha \right)r_{1}]}$, and *L* ≈ 2*Ut*; consequently, we obtain the dimensionless spread radius of the lamella $r_{c}^{*}=r_{c}/r_{2}=r_{1}\theta_{0}/r_{2}\approx \;\sqrt{2tU/[\left(1+1/\alpha \right)r_{2}]}$, where *U* is the impact speed and *r*_c_ is the half arc length of the wetted spherical cap. In previous studies,^[^ ^31^, ^32^, ^33^, ^34^ ^]^ the dimensionless neck radius during droplet impact on a plate was controversially expressed in two forms, $r_{c}^{*}∼\sqrt{2t^{*}}$ and $r_{c}^{*}∼\sqrt{3t^{*}}$, with dimensionless time t* = *tU*/*r*_2_, using either volume conservation or Wagner's theory.^[^ ^35^ ^]^ Here, we observed that the use of a coefficient of three fit our experimental results better, so the final dimensionless spread radius of the lamella $r_{c}^{*}$ in the early stage was rewritten as

(1) $$r_{c}^{*}=\frac{r_{c}}{r_{2}}=\sqrt{\frac{3tU}{\left(1+1/\alpha \right)r_{2}}}$$

To verify the above analysis, systematic experiments were conducted for water droplets impacting hydrophobic/SLIPS spheres with different *α* (Movie S3, Supporting Information). First, as shown in Figure 2b and Movie S4, Supporting Information, a lamella detached more easily from a SLIPS plane than from a hydrophobic plane, similar to Figure 2a. More importantly, in Figure 2d, all curves for the $r_{c}^{*}$ evolution on spheres with or without lubricant films collapse onto a single curve, showing good consistency with our above analysis. Therefore, we successfully unified the lamella dynamics for the water entry of spheres and droplet impact problems through the key parameter of the radius ratio *α*. We also observed that the lamella spreading speed was similar for droplets impacting the hydrophobic and SLIPS spheres, indicating that the presence of a lubricant film has little effect on the early spreading of the lamella.

Next, we probed the critical condition for lamella detachment, an important process related to cavity generation. During impact, a lamella first spreads along the substrate. According to Riboux & Gordillo,^[^ ^34^ ^]^ a lamella can only be ejected if its tip advances faster than *r*_c_, which requires $Du/Dt=-\partial p/\partial x+Re_{d}^{-1}\nabla^{2}u\geq$ *r̎*_c_ for the *x* direction, and the time *t*_e_ for lamella ejection from the neck area can be calculated from the dimensionless form as $c_{1}Re_{d}^{-1}t_{e}^{-1/2}+Re_{d}^{-2}Oh_{d}^{-2}=c^{2}t_{e}^{3/2}$, where *u* is the lamella speed, *p* is the pressure, $c_{1}=\sqrt{3}/2$, *c* = 1.1, *Re*_d_ = *ρ*_l_ *r*_2_ *U*/*μ*_l_, $Oh_{d}=\mu_{l}/\sqrt{\rho_{l}r_{2}\gamma_{l}}$, *ρ*_l_, *r*_2_, *U*, *μ*_l_, and *γ*_l_ are the density, radius, impact velocity, dynamic viscosity, and surface tension of the droplet, respectively. Although this analysis was first performed for droplets impacting solid substrates, Figure 2d reveals the rationality of unifying the early lamella evolution for droplets impact and water entry problems and indicates negligible effects of the lubricant film on $r_{c}^{*}$ for the early stage. Therefore, the dimensionless lamella velocity at the instant of ejection reads $U_{l}/U=\dot{r_{c}^{*}}=\sqrt{3/[\left(1+1/\alpha \right)t_{e}]}/2$.

**Figure 3:**
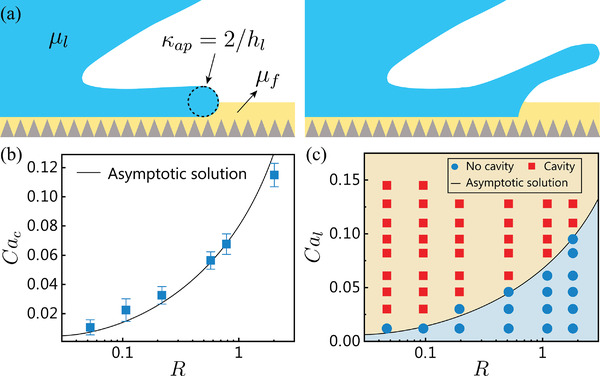
Threshold for lamella detachment and cavity formation. a) Schematic of the dewetting process of a water drop impacting a SLIPS surface with a viscosity ratio *R* = *μ*_l_/*μ*_f_. b) Critical capillary number *Ca*_c_ for lamella detachment in water droplets impacting SLIPS planes. c) Regime map showing the region where cavities were produced, for water entry of SLIPS spheres. Here, silicone oils of different viscosities were used as lubricants. Solid symbols represent the experimental data and the solid line represents our asymptotic solution from Equation (2).

During the prompt spreading of a lamella, if the capillary number *Ca* = *μ*_l_ *u*/*γ*_l_ exceeds the threshold value *Ca*_c_, then the advancing contact line will become unstable, and the lubricant ahead of it will invade into and separate the lamella from the substrate,^[^ ^36^, ^37^ ^]^ termed dewetting. Apparently, we only need to compare *Ca*_l_ = *μ*_l_ *U*_l_/*γ*_l_ with *Ca*_c_ for the dewetting transition, as *u* takes its maximum value *U*_l_ at time *t*_e_. The lamella movement is dominated by the balance between viscous forces and surface tension forces. Following the idea of transformation of interfaces from Qin and Gao,^[^ ^38^ ^]^ we derived an analytical solution of *Ca*_c_ as follows (see Figure S5, Supporting Information and Supporting Information for the detailed derivation)

(2) $$Ca_{c}=\frac{{\tilde{\theta }}_{e}^{3}R}{9}{\left[\ln\left(\frac{Ca_{c}^{1/3}{\tilde{\theta }}_{e}h_{l}}{12^{2/3}\pi \lambda R^{1/3}{\mathrm{Ai}}^{2}\left(t_{\max}\right)}\right)\right]}^{-1}$$

where ${\tilde{\theta }}_{e}={\left[9\int_{0}^{\theta_{e}}\frac{d\theta }{f_{\mathrm{cox}}\left(\theta ;R\right)}\right]}^{1/3}$ is the new interface angle, viscosity ratio *R* = *μ*_l_/*μ*_f_, *μ*_l_, and *μ*_f_ are the dynamic viscosities of the lamella liquid and lubricant (**Figure** **3**a), respectively, $h_{l}\approx \;r_{2}We_{d}^{-1/4}$ is the local lamella thickness,^[^ ^39^, ^40^ ^]^ *λ* is the slip length, *We*_d_ = *ρ*_l_ *r*_2_ *U* ^2^/*γ*_l_, Ai(*t*_1_) is the Airy function of the first kind with the parameter *t*_1_ to be determined by matching the inner solution to the outer solution, and *t*_1_ = *t*_max_ ≈ −1.0188^[^ ^38^ ^]^ is the largest maximum point of Ai(*t*_1_) which corresponds to transition threshold. *κ*_ap_ = 2/*h*_l_ was used as the apparent curvature of the lamella head. The slip lengths *λ* were determined by the best fits to our experimental data, which gave 1.0 × 10^−5^ for water on perfluorinated fluid, and 1.5 × 10^−6^ for water on silicone oils.

The asymptotic solution of *Ca*_c_ is related to the viscosity ratio *R*, and a larger *R* leads to a larger *Ca*_c_. For water droplets impacting hydrophobic surfaces in which the lubricant is air, *R* = *μ*_l_/*μ*_f_ = *μ*_water_/*μ*_air_ = 55.3. In contrast, for water droplets impacting SLIPS surfaces or water entry of SLIPS spheres, in which the lubricant is perfluorinated fluid, *R* = *μ*_water_/*μ*_f_ = 0.67 ≪ 55.3; consequently, the lamella separates much more easily from a SLIPS surface, as demonstrated in Figure 1b,c and Figure 2a,b.

To further verify the dependence of *Ca*_c_ on *R*, a series of water droplets impacting SLIPS plane experiments were conducted by using silicone oils (Clearco Products Co., Inc.) with different viscosities as the lubricant. From the static Neumann conditions, silicone oil will climb up along and finally encapsulate a static water droplet.^[^ ^26^, ^41^ ^]^ Smith et al.^[^ ^26^ ^]^ theoretically derived the initial resistance to movement and the viscous dissipation of a droplet moving on a SLIPS surface, including the cloaking configuration. Keiser et al.^[^ ^42^ ^]^ noted that the lubricant meniscus shape should be speed dependent; therefore, the dependence of friction on the droplet speed should be nonlinear. Later, Keiser et al.^[^ ^43^ ^]^ carried out a comprehensive experimental investigation on viscous dissipation for four distinct regions concerning the oil foot around a moving droplet and obtained a compact universal friction scaling law as the droplet velocity to a power of 2/3. Kim and Rothstein^[^ ^44^ ^]^ investigated the effects of *R* on droplet impact dynamics and observed an increase in the droplet spreading rate and the maximum spread diameter as the lubricant viscosity decreased when the droplet had a larger viscosity than the lubricant. Nevertheless, as shown in Figure 2d, we did not observe evident differences in $r_{c}^{*}$ for droplets impacting in different configurations, including the 0.65 cSt silicone oil‐infused surface (less viscous than water), indicating ignorable effects of the lubricant viscosity and climbing of the lubricant in the very early lamella evolution. As shown in Figure 3b (Movie S5, Supporting Information), the experimentally measured *Ca*_c_ well matches our asymptotic solution for over 1.5 orders of magnitude of *R*. Similarly, Figure 3c (Movie S6, Supporting Information) shows a comparison between the experimentally determined occurrence of cavity formation for water entry of SLIPS spheres with silicone oils of different viscosities as the lubricant and *Ca*_c_ calculated by solving Equation (2). Herein, the red squares denoting cavity formation represent smooth cavities formed and sustained after impact, as shown on the right side of Figure 1c. Figure 3c shows that all minima of *Ca*_l_ for cavity formation take slightly larger values than *Ca*_c_ for different *R*, which is understandable, as a stable cavity not only requires lamella detachment (*Ca*_c_) but also sufficient air entrainment. The water spray emerging from splash crown closure, termed a surface seal, could also impede the formation of a smooth and stable cavity.^[^ ^11^ ^]^

**Figure 4:**
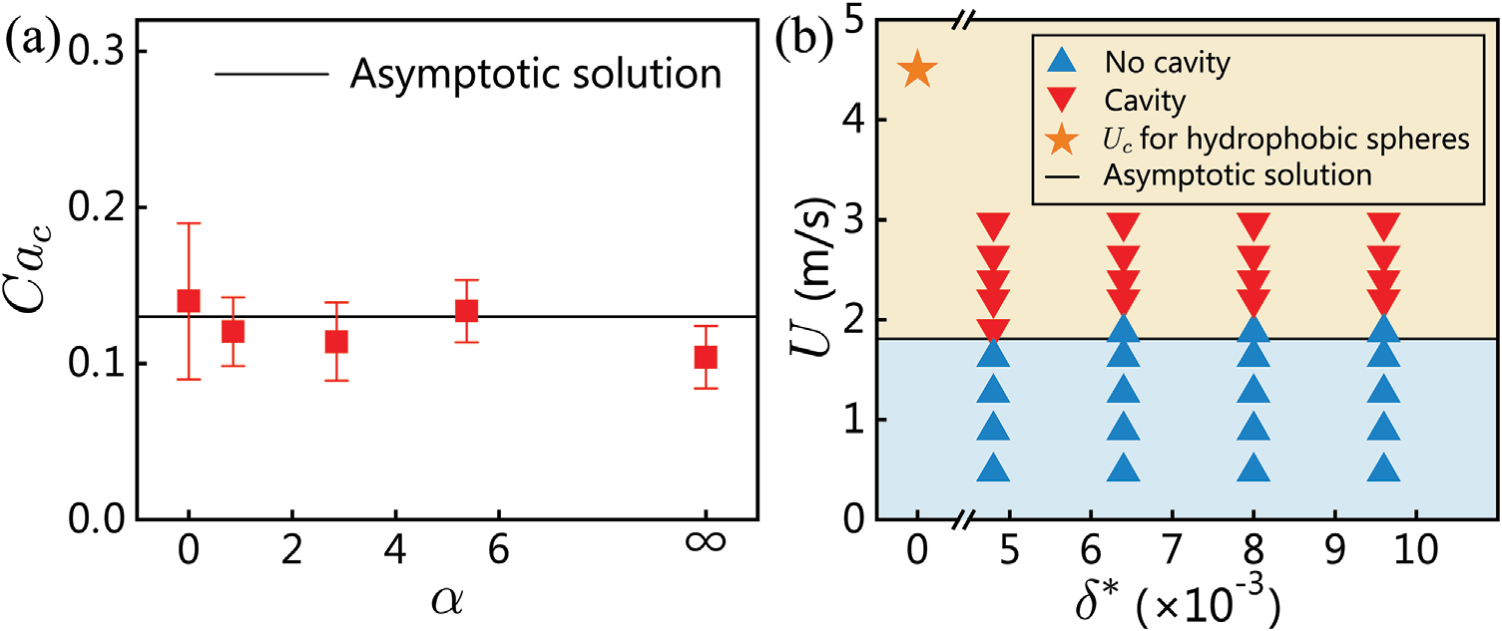
Effects of α and dimensionless lubricant layer thickness δ* on cavity formation. a) Negligible dependence of *Ca*_c_ on *α*. b) Inconspicuous change in threshold impact speed *U* for cavity formation, with a two times change in dimensionless lubricant layer thickness δ* in the water entry of hydrophobic and SLIPS spheres. Perfluorinated fluid FC‐3283 was used as the lubricant.

Regarding the effect of radius ratio α, **Figure** **4**a plots the *Ca*_c_ for water droplets impacting perfluorinated fluid‐infused SLIPS spheres with different radii. The experimentally measured values well match the asymptotic solution across the range of α from 0 (water entry) to ∞ (droplet impact), reconfirming our deduction that the lamella dynamics for water entry of spheres and droplet impact problems can be unified with α.

In addition, we briefly discuss the influence of lubricant thickness on cavity formation in the water entry of SLIPS spheres. As previously mentioned, sufficient lubricant was added to the textured sphere, giving a lubricant film with thickness *δ*_f_ > 25 µm, corresponding to a dimensionless thickness δ* = *δ*_f_/*r*_1_ > 5 × 10^−3^. The apparent contact angle hysteresis measured was less than 5° (Movie S7, Supporting Information). Figure 4b shows that the dependence of threshold velocity *U*_c_ for cavity formation on δ* is inconspicuous for a twofold change in δ* in our experiment. However, the water entry of a SLIPS sphere requires a much lower *U*_c_ ≈ 1.8 m s^−1^ than that for a hydrophobic sphere with the same contact angle (≈4.5 m s^−1^).

**Figure 5:**
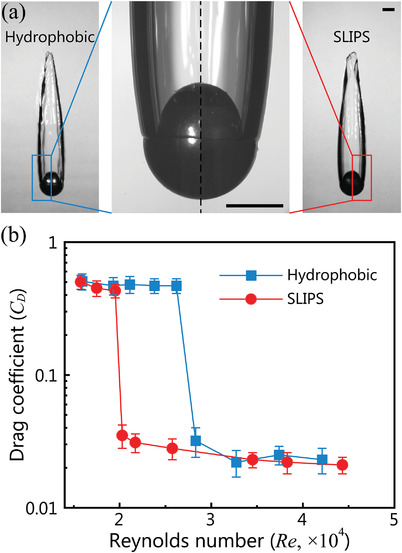
Cavity formation and drag reduction performance of hydrophobic and SLIPS spheres in water. a) Cavity configurations of hydrophobic and SLIPS spheres in water. The two spheres have the same impact speed of 4.5 m s^−1^, and reach the same terminal speed of 1.77 m s^−1^. The scale bars are 5 mm. b) Relation between the drag coefficient *C*_D_ and the Reynolds number *Re* for hydrophobic and SLIPS spheres in water.

Drag reduction for a moving solid in a liquid is crucial for energy savings.^[^ ^9^, ^16^, ^17^, ^45^ ^]^ Urgent needs are found in military scenarios such as underwater missile launches and air launches of torpedoes from warplanes. It is shown that a moving sphere underwater without a cavity usually experiences a larger drag than in cavity‐forming cases due to the shedding of a strong, ring‐like vortex structure.^[^ ^46^ ^]^ Vakarelski et al. demonstrated that a sphere surrounded by a cavity could achieve a 90% drag reduction as compared to a sphere without a cavity.^[^ ^9^ ^]^ They also showed that the self‐determined shapes of the gas cavities could be predicted by the potential flow theory including gravity. For a hydrophobic or SLIPS sphere in our case, we did not obtain a cavity which could fully surround the sphere, as evident by the existence of a contact line near the equator of the sphere (Figure 5a). Nevertheless, our numerical results show that the pressure distribution along the cavity surface does obey Bernoulli's equation (Figure S7, Supporting Information). Finally, to elucidate the potential application of this intriguing interfacial effect, we investigated the change in the drag coefficient with the Reynolds number in the water entry of hydrophobic and SLIPS spheres at the same contact angle. **Figure** **5**a demonstrates that the cavity configurations and contact line positions for hydrophobic and SLIPS spheres are identical for the same impact velocity of ≈4.5 m s^−1^ (Movie S8, Supporting Information). We quantified the drag coefficient of the two spheres through their time‐dependent average velocities and cavity volumes by profile‐matching and image processing methods (Figure S8, Supporting Information). For a sphere moving at a fixed velocity *U* in a liquid, the drag coefficient *C*_D_ yields Equation (3)^[^ ^3^, ^9^, ^15^, ^19^, ^46^ ^]^

(3) $$C_{D}=\frac{8gM_{\text{eff}}}{\rho \pi D_{\text{eff}}^{2}U^{2}}$$

where *g* is the gravitational acceleration, *M*_eff_ = *m*_s_ − *ρ* *V*_c_ is the effective mass of a sphere‐in‐cavity structure, *m*_s_ is the sphere mass, *ρ* is the liquid density, *V*_c_ is the cavity volume including the sphere, and *D*_eff_ is the effective sphere diameter. As sketched in Figure 5b, despite the similar values of *Re* = *ρ* *UD*_eff_/μ observed in the later stage (*Re* ≳ 3 × 10^4^), the drastic decline in *C*_D_ appeared much earlier for the SLIPS sphere, indicating its better underwater drag reduction effect compared to that of the hydrophobic sphere at lower impact velocities. Notably, for superhydrophobic spheres, the critical velocity for creating a cavity is extremely low;^[^ ^14^ ^]^ consequently, the drag reduction performance of superhydrophobic spheres will be superior. Nevertheless, the highlight of our work is the capacity to reduce drag at a lower Reynolds number for a relatively small contact angle (≈110°).

## 3 Conclusion

To conclude, we have demonstrated a new approach to form robust gas cavities for water entry of spheres. The simple infusion of a textured solid body with a uniform and stable lubricant layer can produce and sustain stable gas cavities at lower impact velocities compared to solid counterparts with similar wettability. Relying on our first unification of the lamella dynamics for water entry of spheres and droplets impacting planes through the key parameter of the radius ratio, we rationalize such phenomena by the preferential lamella detachment from the SLIPS surfaces due to the higher viscosity of the lubricant relative to air. This study should bring new physical concepts regarding conventional water entry issues, which may also benefit engineering applications such as drag reduction and energy savings for underwater moving projectiles.

## 4 Experimental Section

### Spheres and Liquids

The solid spheres used in this paper were made of steel (density *ρ* = 7800 kg m^−3^, diameter *D* = 3, 7, and 10 mm). The properties of the different liquids used in the experiments were taken from the manufacturers' information. Deionized water with a resistivity of 18.3 MΩcm was used for the measurements. The physical properties of the other liquids used in the experiment are shown in **Table** **1**.

**Table 1:** Physical property of the liquids used in the experiment at 20 °C

	Density	Surface	Dynamic
	*ρ*	tension *γ*	viscosity *μ*
	[kg m^−3^]	(mN m^−1^]	[mPa s]
Air	1.20	—	0.018
Water	998	72.9	1.0
FC‐3283	1830	16.0	1.5
0.65 cSt silicone oil	761	15.9	0.49
1 cSt silicone oil	818	17.4	0.82
1.5 cSt silicone oil	851	18.0	1.3
2 cSt silicone oil	873	18.7	1.7
5 cSt silicone oil	918	19.7	4.6
10 cSt silicone oil	935	20.1	9.4
20 cSt silicone oil	950	20.6	19

### Fabrication and Characterization

Before femtosecond laser irradiation, the steel spheres were ultrasonically cleaned in acetone, isopropanol, ethanol, and deionized water, respectively. The cleaned samples were mounted on a mobile platform and then irradiated by a femtosecond laser using vertically crossed line‐by‐line scanning. Laser pulses (central wavelength of 800 nm, 1 kHz train of 104 fs) from a regeneratively amplified Ti:sapphire femtosecond laser system (Coherent, Legend Elite‐1K‐HE, USA) were employed for irradiation. The laser beam was focused onto the sphere surface and scanned along the *x–y* coordinate directions through a galvanometric scanning system (ScanLab, Germany) equipped with a telecentric *f*–*θ* lens with a focal length of 63 mm. The scanning spacing between two adjacent lines was 15 µm in both the *x* and *y* coordinate directions. The laser mean power was measured as 360 mW on a 20 µm spot (≈1.15× 10^5^ W cm^−2^). The sphere surface to be scanned was divided into a series of adjacent quasi‐square subareas with a side length of ≈2 mm each, and the scanning speed was set in the range of 1 to 5 mm s^−1^. After laser ablation, the laser‐textured spheres were first immersed into 3% 1H, 1H, 2H, 2H‐perfluorodecyltriethoxysilane‐ethanol solution for 24 h and then heated in a drying oven at 120 °C for 30 min to form superhydrophobic spheres. To fabricate SLIPS spheres, the superhydrophobic spheres were infused with a certain amount of perfluorinated fluid (3M Fluorinert FC‐3283) on the microstructured surfaces via drop coating method. To fabricate hydrophobic spheres, the cleaned spheres were coated with an electronic grade coating (3M Novec 1700) at room temperature (20 °C). The surface morphologies of the laser‐textured spheres were observed by a secondary electron scanning electron microscope (Zeiss EVO18) at an accelerating voltage of 10 kV.

### Impact Experiments

For water entry experiments, spheres were released from rest at varying heights above a transparent acrylic vessel containing the test liquid. For drop impact experiments, droplets were produced from a hollow glass capillary (outer diameter of 1.5 mm and inner diameter of 0.86 mm), and the flux was controlled by an injection pump (Fusion 200, Chemyx Inc., USA). The diameter and impact speed were calculated from the high‐speed recorded images. The impact process was recorded using a high‐speed video camera (Phantom V2512 or VEO 710S) at typical rates from 7500 to 110 000 fps. Each release was carried out under atmospheric pressure at room temperature.

### Statistical Analysis

To find an accurate threshold for cavity formation, each experiment was repeated five times in the vicinity of the critical velocity. Snapshots of experiments were analyzed to measure physical quantities such as velocity and cavity volume by profile matching and edge detection with the commercial software Solidworks and MATLAB. The accuracy in analysis was one pixel, which corresponded to 3.5–166.7 µm, depending on different magnifications used. Wolfram Mathematica was used to calculate the numerical integration in the curvature transformation and solve the implicit equation of the critical capillary number. The finite element software FreeFem++ was used to solve the Laplace equation of the velocity potential under the inviscid assumption.

## Conflict of Interest

The authors declare no conflict of interest.

## Supporting information

Supporting Information

Supplemental Movie 1

Supplemental Movie 2

Supplemental Movie 3

Supplemental Movie 4

Supplemental Movie 5

Supplemental Movie 6

Supplemental Movie 7

Supplemental Movie 8

## Data Availability

The data that support the findings of this study are available from the corresponding author upon reasonable request.
