# Supplementary material for: Sustaining Robust Cavities with Slippery Liquid–Liquid Interfaces
Source: Adv Sci (Weinh). 2022 Jan 17;9(7):2103568. doi: 10.1002/advs.202103568 (PMC8895157; doi:10.1002/advs.202103568)
Supplement: Supplementary file 1 — Supporting Information [file ADVS-9-2103568-s008.pdf]

ADVANCED SCIENCE
Open Access

# Supporting Information

for *Adv. Sci.*, DOI 10.1002/advs.202103568

Sustaining Robust Cavities with Slippery Liquid–Liquid Interfaces

*Suwan Zhu, Tao Wu, Yucheng Bian, Chao Chen, Yiyuan Zhang, Jiawen Li, Dong Wu*, Yanlei Hu, Jiaru Chu, Erqiang Li* and Zuankai Wang**

ADVANCED SCIENCE
Open Access

# Supporting Information

for *Adv. Sci.,* DOI: 10.1002/advs.202103568

# Sustaining robust cavities with slippery liquid-liquid interfaces

*Suwan Zhu, Tao Wu, Yucheng Bian, Chao Chen, Yiyuan Zhang, Jiawen Li, Dong Wu,* Yanlei Hu, Jiaru Chu, Erqiang Li,* Zuankai Wang,**

# Sustaining robust cavities with slippery liquid-liquid interfaces

*Suwan Zhu*[1] *Tao Wu*[2] *Yucheng Bian*[1] *Chao Chen*[1] *Yiyuan Zhang*[1] *Jiawen Li*[1] *Dong Wu*[1,*] *Yanlei Hu*[1] *Jiaru Chu*[1] *Erqiang Li*[2,*] *Zuankai Wang*[3,4,*]
[1]CAS Key Laboratory of Mechanical Behavior and Design of Materials, Hefei National Laboratory for Physical Sciences at the Microscale, Laboratory of Precision Scientific Instrumentation of Anhui Higher Education Institutes, Department of Precision Machinery and Precision Instrumentation, University of Science and Technology of China, Hefei 230026, China.
[2]Department of Modern Mechanics, University of Science and Technology of China, Hefei 230026, China.
[3]Department of Mechanical and Biomedical Engineering, City University of Hong Kong, Hong Kong, China.
[4]Shenzhen Research Institute of City University of Hong Kong, Shenzhen 518057, China.

Corresponding authors:
Prof. Dong Wu, dongwu@ustc.edu.cn
Prof. Erqiang Li, eqli@ustc.edu.cn
Prof. Zuankai Wang, zuanwang@cityu.edu.hk

Dr. Suwan Zhu and Tao Wu contributed equally to this work.

Keywords: *slippery surfaces, drag reduction, cavity formation, water entry, droplet impact*

## 1 Materials.

The properties of the different liquids used in the experiments were taken from the manufacturers (see Table 1 in the main text). Deionized water with a resistivity of 18.3 MΩ·cm was used for the experiments.

## 2 Sample Fabrication and Preparation.

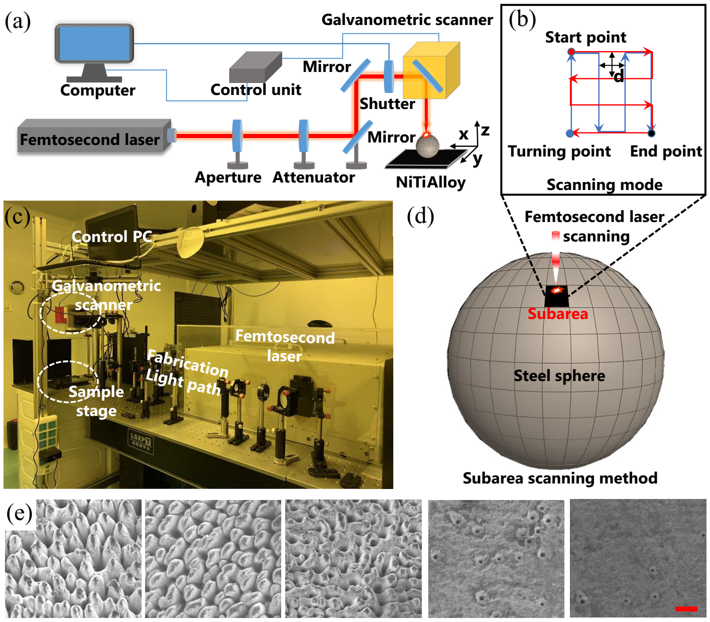


Figure S1: Femtosecond laser fabrication of textured spheres. (a) Optical pathway diagram in textured spheres fabrication via femtosecond-laser irradiation. (b) Schematic of laser scanning path. The steel sphere is ablated by vertically crossed line-by-line laser scanning. (c) Laser fabrication system consists of a femtosecond laser (Coherent, Legend Elite-1K-HE, USA), a fabrication light path, a galvanometric scanner (ScanLab, Germany), a sample stage and a control PC. (d) Subarea scanning method. The sphere surface to be scanned is divided into a series of adjacent quasi-square subareas with a side length of ~ 2 mm for each. When one subarea scanning ends, another adjacent subarea will be moved to the laser-focused point to continue scanning until finishing the hole surface coverage. (e) Dependence of surface morphology on laser scanning speed. The average depth of microstructures was about 50, 32, 24, 8 and 2 $\mu$m respectively, relative to scanning speed 1 to 5 mm/s respectively. Scale bar is 30 $\mu$m.

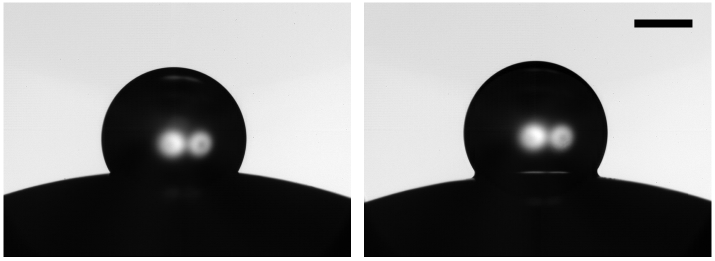

Figure S2: Water contact angle of hydrophobic sphere (left) and SLIPS sphere (right). Here the contact angles were measured through the static sessile drop method[1] with a droplet volume of 0.9 $\mu$L. Scale bar is 500 $\mu$m.

## 3 Experiments.

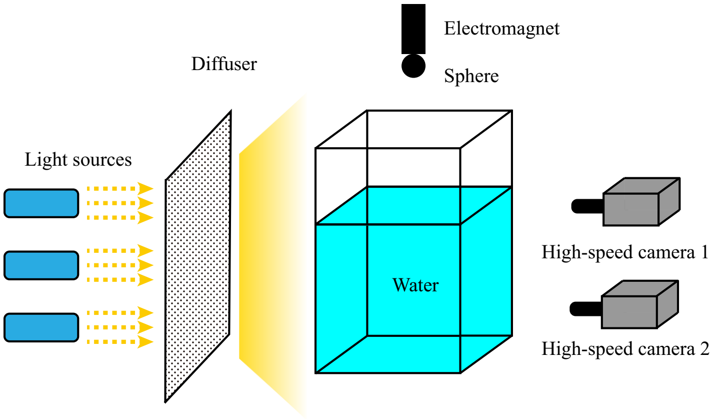


Figure S3: Sketches of the experimental set-ups. The system consists of two high-speed video cameras (Phantom v2512 and Phantom VEO 710S), an electromagnetic release system, three 350 W metal-halide lamps (Sumita) and a water tank. The sphere is released via the electromagnetic release system equipped with a sample holder at any desired heights. The top and bottom cameras are used to record interface evolution near and below the air-water surface, respectively.

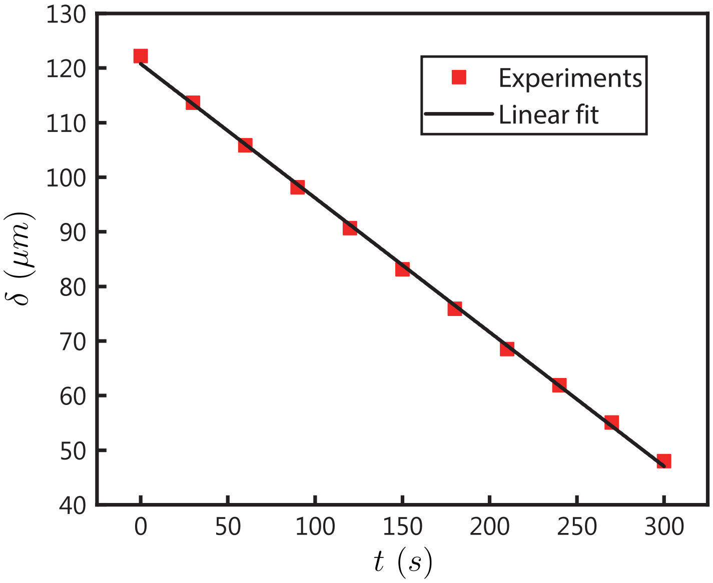


Figure S4: Evaporation of lubricant on a planar SLIPS surface in air. The initial lubricant film thickness $\delta$ is set to be $\sim 120$ $\mu$m. The film thickness decreases with time, giving an evaporation rate nearby 0.3 $\mu$m/s in air.

For water entry experiments, sphere was attached to an electromagnetic release system positioned directly above the center of a water tank. After adding the lubricant to the hanging sphere via a pipette, the sphere was released and the rapid surface motions during the impact were recorded by two high-speed cameras (Phantom v2512 or Phantom VEO 710S), as shown in Figure S3. The lubricant evaporation rate was evaluated by measuring the mass loss from a planar SLIPS surface, which gave a decrease in

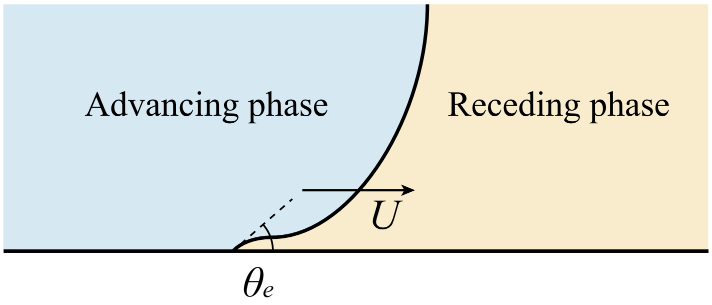


Figure S5: Schematic of advancing contact line geometry.

lubricant film thickness of $\sim 0.3$ $\mu$m/s (Figure S4). Therefore, lubricant evaporation is negligible during the experimental operation.

For droplets impact experiments, the droplets were squeezed out from a hollow glass capillary (outer diameter of 1.5 mm and inner diameter of 0.86 mm). The droplet diameter and impact speed were calculated from the high-speed recorded images. The impact process was recorded using a high-speed video camera Phantom v2512 at typical rates from 7,500 to 110,000 fps.

## 4 Derivation of $Ca_c$.

Following the idea of transformation of interfaces by Qin & Gao [2], a dynamic wetting flow with arbitrary slope angle and viscosity ratio can reduce to a small-slope problem of receding contact lines. The new interface slope is determined as

$$\theta_e = \left[ 9 \int_0^{\theta_e} \frac{\mathrm{d}\theta}{f_{cox}(\theta; R)} \right]^{1/3}, \tag{1}$$

where the function $f_{cox}(\theta; R)$ is a complicated function given by Cox [3] and Chan [4], taking the following form:

$$\begin{aligned} f_{cox}(\theta; R) &\equiv \frac{2\sin^3\theta\left[R^2 f_1(\theta) + 2R f_3(\theta) + f_1(\pi-\theta)\right]}{3[R f_1(\theta) f_2(\pi-\theta) - f_1(\pi-\theta) f_2(\theta)]}, \\ f_1(\theta) &\equiv \theta^2 - \sin^2\theta, \\ f_2(\theta) &\equiv \theta - \sin\theta\cos\theta, \\ f_3(\theta) &\equiv \left(\theta(\pi-\theta) + \sin^2\theta\right). \end{aligned} \tag{2}$$

Here, the contact angle $\theta_e$ is defined using the parameters of receding phase, as shown in Figure S5. The viscosity ratio is $R = \eta_{adv}/\eta_{rec}$, where $\mu_{rec}$ and $\mu_{adv}$ are the viscosity of the receding and advancing phase, respectively.

When a liquid is forced to rapidly spread on a solid surface, the capillary force fails to compete

with the viscous drag, leading to the entrainment of gas [5]. The competition between capillary force and viscous force is characterized by a dimensionless capillary number $Ca = \mu_{rec}U/\gamma$ where $\gamma$ is the surface tension. Note that $Ca$ is defined using receding phase viscosity the capillary number based on advancing phase viscosity is simply $CaR$. The critical $Ca_t$ of dewetting process was derived by asymptotic method under lubrication approximation:

$$\left.\begin{aligned} \tilde{\theta}_{ap} &= -\frac{(12Ca_t)^{1/3}\mathrm{Ai}'(t_1)}{Ai(t_1)}, \\ \frac{\kappa_{ap}}{2}\frac{\tilde{\theta}_{ap}\tilde{\theta}'_{ap}}{\sin\theta_{ap}} &= \frac{Ca_t^{1/3}\tilde{\theta}_e \exp\left[-\tilde{\theta}_e^3/(9Ca_t)\right]}{12^{2/3}\pi\lambda \mathrm{Ai}^2(t_1)}, \end{aligned}\right\} \tag{3}$$

where $\kappa_{ap} = 2/h_l$ in our experiments, $\lambda$ is the slip length, and $\mathrm{Ai}(t_{max})$ is the Airy function of the first kind with the matching parameter $t_{max} \approx$ -1.0188. Based on advancing phase viscosity, the critical capillary number $Ca_c = Ca_t R$ of dewetting process is

$$Ca_c = \frac{\tilde{\theta}_e^3 R}{9}\left[\ln\left(\frac{Ca_c^{1/3}\tilde{\theta}_e h_l}{12^{2/3}\pi\lambda R^{1/3}\mathrm{Ai}^2\left(t_{\max}\right)}\right)\right]^{-1} \tag{4}$$

## 5 Potential theory

In our experiments, the Weber number is of $\sim$ 1000 and the Reynolds number is of $\sim$ 20000, indicating that the effect of viscous force and surface tension is negligible, and the dynamic evolution is dominated by inertia and gravity. For the no-cavity regime, previous study[1] has shown that the vortex shedding and pressure pulsation behind the sphere will reduce the moving velocity. For the cavity-forming regime, the presence of cavity will mitigate vortex shedding and effectively produces a potential-flow-like field, thereby reducing the drag coefficient of underwater motion. Specifically, when the sphere reaches the terminal velocity, the incompressible steady Bernoulli equation will be satisfied along the cavity surface, which reads

$$\frac{1}{2}u^2 + \frac{p}{\rho} + gz = \text{constant}, \tag{5}$$

where $u$ is the velocity, $p$ is the pressure, $\rho$ is the density, $g$ is the gravitational acceleration and $z$ is the relative height.

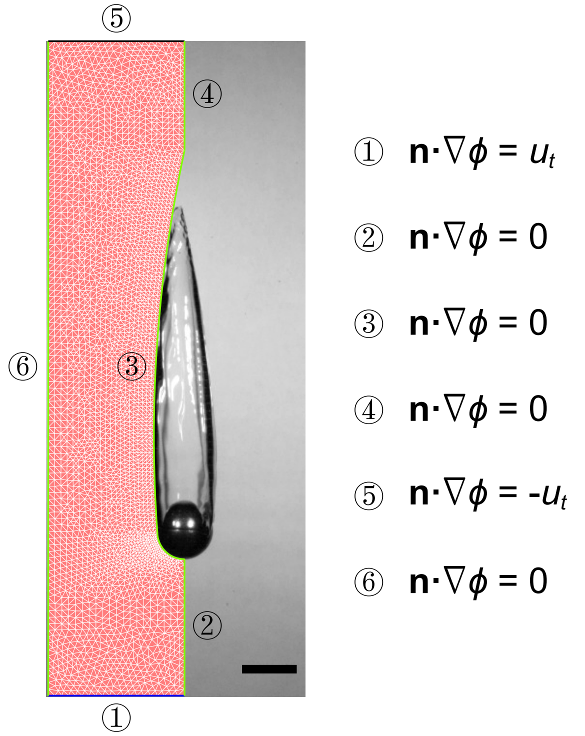


Figure S6: Mesh partition and boundary conditions for finite element calculation of velocity potential $\varphi$. The scale bar is 10 mm.

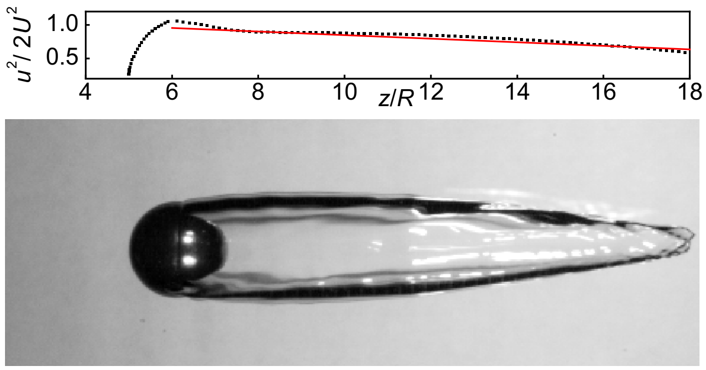


Figure S7: Dynamic pressure distribution on cavity surface.

To verify above analysis, the finite element method was employed to calculate the pressure distribution along the cavity surface. For an irrotational flow $\nabla \times \mathbf{u} = \mathbf{0}$, a scalar velocity potential $\varphi$ can be introduced as $\mathbf{u} = \nabla\varphi$. With the incompressibility condition $\nabla \cdot \mathbf{u} = \mathbf{0}$, the velocity potential will satisfy the Laplace equation $\nabla^2\varphi = 0$. We extracted the boundary of the cavity in the experiment, and analyzed the pressure distribution on it using the open source finite element software freefem++. The grid division and boundary condition settings are shown in Fig. S6, where the velocities of top and bottom boundaries were fixed as terminal velocity 1.77 m/s, and the other boundaries satisfied the normal non-penetration condition. Figure S7 shows a linear relationship between the dynamic pressure and the

relative height, which is in consistent with our inference. Once the cavity is formed, the potential-flow-like streamlined configuration of it can effectively reduce the drag.

## 6 Profile-matching and image processing methods.

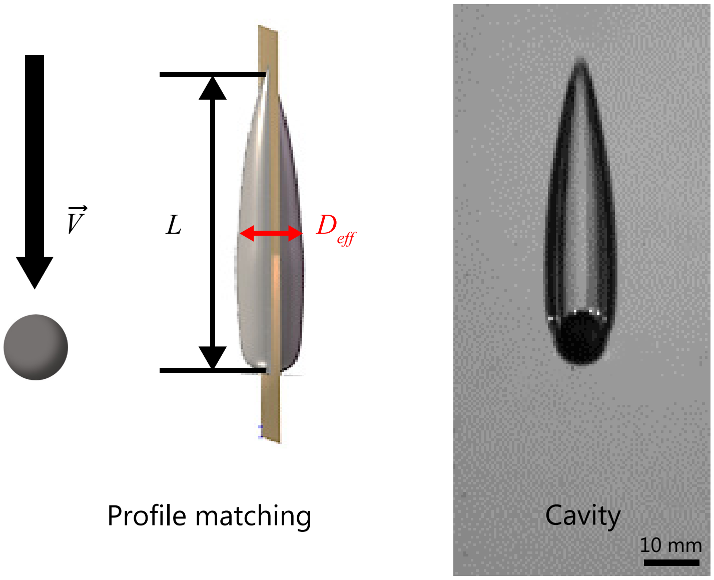


Figure S8: High-resolution snapshots of the sphere-in-cavity structure were analyzed to estimate the effective sphere diameter $D_{eff}$ and volume $V_c$ by profile-matching and image processing methods.

The key to calculate the Reynolds number $Re = \rho U D_{eff}/\mu$ for the spheres moving in liquids is determining the effective sphere diameter $D_{eff}$ and the effective mass of sphere-in-cavity structure ($M_{eff} = m_s - \rho V_c$). Accordingly, high-resolution snapshots of the sphere-in-cavity structure were analyzed to estimate $D_{eff}$ and $V_c$ by profile-matching and imaging processing, which were achieved with the commercial software such as Solidworks$^{\mathrm{TM}}$ and MATLAB$^{\mathrm{TM}}$. As shown in Figure S8, we calculated the Reynolds number for moving spheres by extracting the effective sphere diameter $D_{eff}$ and the sphere-in-cavity structures volume $V_c$.

## References

[1] T. T. Truscott, B. P. Epps, A. H. Techet, *J. Fluid Mech.* **2012**, *704* 173.

[2] J. Qin, P. Gao, *J. Fluid Mech.* **2018**, *844* 1026.

[3] R. G. Cox, *J. Fluid Mech.* **1986**, *168* 169.

[4] T. S. Chan, S. Srivastava, A. Marchand, B. Andreotti, L. Biferale, F. Toschi, J. H. Snoeijer, *Phys. Fluids* **2013**, *25* 074105.

[5] J. H. Snoeijer, B. Andreotti, *Annu. Rev. Fluid Mech.* **2013**, *45* 269.
